# Quorum Sensing Controls Swarming Motility of *Burkholderia glumae* through Regulation of Rhamnolipids

**DOI:** 10.1371/journal.pone.0128509

**Published:** 2015-06-05

**Authors:** Arvin Nickzad, François Lépine, Eric Déziel

**Affiliations:** INRS—Institut Armand-Frappier, Laval, Québec, Canada; Ben-Gurion University of the Negev, ISRAEL

## Abstract

*Burkholderia glumae* is a plant pathogenic bacterium that uses an acyl-homoserine lactone-mediated quorum sensing system to regulate protein secretion, oxalate production and major virulence determinants such as toxoflavin and flagella. *B*. *glumae* also releases surface-active rhamnolipids. In *Pseudomonas aeruginosa* and *Burkholderia thailandensis*, rhamnolipids, along with flagella, are required for the social behavior called swarming motility. In the present study, we demonstrate that quorum sensing positively regulates the production of rhamnolipids in *B*. *glumae* and that rhamnolipids are necessary for swarming motility also in this species. We show that a *rhlA*- mutant, which is unable to produce rhamnolipids, loses its ability to swarm, and that this can be complemented by providing exogenous rhamnolipids. Impaired rhamnolipid production in a quorum sensing-deficient *B*. *glumae* mutant is the main factor responsible for its defective swarming motility behaviour.

## Introduction

*Burkholderia glumae* is a notorious phytopathogen of rice that uses a LuxI/LuxR-type quorum sensing (QS) system to regulate diverse cellular processes such as protein secretion, oxalate production and expression of major known virulence factors, including toxoflavin, lipase, KatG and flagella [1–4]. In *B*. *glumae*, the TofR QS regulator is activated by the *N*-octanoyl homoserine lactone (C_8_-HSL) autoinducer, the product of the TofI *N*-acyl homoserine lactone synthase, to control the expression of the target genes [5].

Among major virulence determinants of *B*. *glumae*, flagellum-driven motility plays a critical role in its pathogenicity [1]. Besides swimming motility, this bacterium is also able to spread by a social motility behaviour called swarming motility, which typically requires both functional flagella and production of a surface-wetting agent (surfactant) [6]. Since swarming is a multicellular behaviour, in many bacteria it is dependent on QS regulation [7]. QS modulates swarming motility by diverse mechanisms, either by regulating the flagellar machinery and propulsive force, or by inducing the expression of wetting agent biosynthesis pathways [8]. For instance, in *B*. *glumae*, QS positively regulates the expression of flagellar biosynthesis genes, which are essential for swarming motility [1]. Furthermore, in *B*. *glumae*, growth temperature also regulates flagellar morphogenesis [9]. Indeed, while a QS-deficient mutant is aflagellated at 37°C and thus does not exhibit swarming nor swimming motilities, it retains normal flagella and motility at 28°C, which shows that flagellum formation requires QS at 37°C but not at 28°C [1]. The wetting agent allowing swarming motility of *B*. *glumae* has not been identified yet. In some swarming bacteria, such as *Pseudomonas aeruginosa* and *Burkholderia thailandensis*, this social motility behaviour is promoted by the production of rhamnolipids [10–12]. We have previously reported that *B*. *glumae* also is capable of rhamnolipids production and carries a *rhl* operon homologous to those responsible for rhamnolipid biosynthesis in *B*. *thailandensis*, *Burkholderia pseudomallei* and *P*. *aeruginosa* [13].

Here we investigate the regulation and role of rhamnolipids in *B*. *glumae*. We confirm that a *rhlA* ^*-*^ mutant which lacks rhamnolipid production completely loses its ability to swarm. Furthermore, we show that QS positively regulates rhamnolipid production, and thus swarming motility. Importantly, although QS is necessary for flagellar motility at 37°C, our results demonstrate that the observed lack of swarming at lower temperatures solely goes through QS-dependent regulation of rhamnolipid production. Given that QS regulates many virulence determinants in *B*. *glumae*, such as flagellar motility, rhamnolipids, as factors involved in surface-associated motility might contribute to the pathogenicity of this bacterium.

## Materials and Methods

### Bacterial strains, plasmids, media and growth conditions

All *B*. *glumae* strains used are derivatives of the wild-type strain BGR1 and are listed in Table 1. Unless otherwise specified, the bacteria were routinely grown from frozen stocks by culturing at 37°C in tryptic soy broth (TSB) (BD) and 240 rpm in a TC-7 roller drum (New Brunswick, Canada), or on TSB agar plates. Antibiotics were used at the following concentrations: tetracycline (Tc), 10 μg ml^-1^, gentamycin (Gm), 20 μg ml^-1^ and triclosan 25 μg ml^-1^.

**Table 1:** Bacterial strains and plasmids.

Bacteria	Relevant characteristics	Source or reference
***E*. *coli***		
DH5α	F-, φ80d*lacZ*ΔM15, Δ(*lacZYA-argF*)U169, *deoR*, *recA*1, *endA*1, *hsdR*17(rk-, mk+), *phoA*, *supE*44, λ-, *thi*-1, *gyrA*96, *relA*1	[14]
SM10 λ*pir*	thi-1 *thr leu tonA lacY supE recA*::RP4-2-Tc::Mu Km^r^ λpir	[15]
***B*. *glumae***		
BGR1	Wild-type, Rif^R^	[16]
BGS2	*tofI*::Ω in BGR1	[5]
ED2106	Deletion of *rhlA* in BGR1	This study
ED2123	BGR1::pAN2	This study
ED2124	BGS2::pAN2	This study
**Plasmids**		
pEX18Gm	Gm^r^, *oriT*+ *sacB*+, gene replacement vector with multiple cloning site from pUC18	[17]
pFTC1	Tetracycline resistance cassette FRT vector, Tet^R^	[18]
pFLPe4	Curable Flp recombinase expression vector, Km^R^	[19]
mini-CTX-*lux*	Integration vector with promoterless *luxCDABE*	[20]
pAN1	pEX18Gm with 5’ and 3’ fragments of *rhlA* interrupted by a Tc^R^ cassette	This study
pAN2	*rhlA* promoter fused to *luxCDABE* in mini-CTX-*lux*	This study

For swarming and swimming assays, plates consisted of modified M9 medium [20 mM NH_4_Cl; 12 mM Na_2_HPO_4_; 22 mM KH_2_PO_4_; 8.6 mM NaCl; 1 mM MgSO_4_; 1 mM CaCl_2_·2H_2_O; 11 mM dextrose; 0.5% casamino acids (Difco)], solidified with 0.5% or 0.25% Bacto-agar (Difco) respectively [11]. The method was essentially as before [21], except that plates were allowed to dry for 30 min, and 34°C was chosen as the incubation temperature. These key parameters which affect swarming were optimized for *B*. *glumae* in order to maximize the reproducibility of swarm plate results. One ml of an overnight culture of each strain was adjusted to OD_600_ 3.0, centrifuged and washed twice with phosphate-buffer saline (PBS) and suspended in 100 μl PBS. To inoculate the plates, 5 μl (swarming) or 1 μl (swimming) of bacteria suspension were spotted in the center. Five replicates were performed for every test and the experiments were performed three times, independently.

The same medium as above without agar was used for production of rhamnolipids in liquid cultures. To determine whether rhamnolipid production could be restored by exogenous addition of signal molecules, culture media were supplemented with 8 μM C_8_-HSL (Sigma-Aldrich). The extract of *B*. *glumae* rhamnolipids used for complementation of swarming was obtained as described previously [22].

### Mutant and reporter construction

Plasmids utilized are listed in Table 1. A *rhlA* ^*-*^ mutant was generated by allelic replacement with a fragment of gene bglu_2g05650 (*rhlA*) containing a Tc^R^ resistance cassette flanked by FLP recognition target (FRT) sites. To accomplish this, a 862 bp DNA fragment corresponding to the 5’ end of *rhlA* and adjacent chromosomal sequences was amplified using primers VDrhlA2F and VDrhlA2R, and a 763 bp DNA fragment corresponding to the 3’ end of *rhlA* and its adjacent sequences was amplified using VDrhlA3F and VDrhlA3R (Table 2). A 2014 bp Tc^R^ resistance cassette was amplified from the pFTC1 plasmid with 50-bp overhang sequences identical to the adjacent region fragments by using primers VDrhlA1F and VDrhlA1R. In a second amplification, the three PCR products were joined together and amplified using the primer pair VDrhlA2F/VDrhlA3R. The resulting 3639 bp PCR product was T/A cloned into the pGEM-T Easy Vector (Promega, Madison, WI, USA), excised by *Eco*RI digestion and finally ligated into the corresponding site of pEX18Gm [17]. This construct, pAN1, was introduced into *B*. *glumae* BGR1 by conjugation with donor *E*. *coli* SM10λpir and the recombinants were selected on LB agar with Tc (10 μg ml^-1^) while *E*. *coli* was inhibited with triclosan (25 μg ml^-1^). To counterselect for bacteria that had undergone a double crossover event, colonies were streaked onto LB agar supplemented with sucrose (30%) and Tc (10 μg ml^-1^). Gm-sensitive colonies were analyzed by PCR to verify that *rhlA* had been successfully replaced with a mutated copy of the allele. Finally, the Tc^R^ cassette was excised by Flp-mediated recombination, using pFLPe4 [19]. The Flp-mediated removal of the Tc^R^ cassette was confirmed by PCR.

**Table 2:** PCR Primers.

Oligonucleotides	Sequence (5’→ 3’)
VDrhlA2F	CCTGGAATTTTTCCCGTTTT
VDrhlA2R	CAGGTCGAAGCAGATGGAAT
VDrhlA3F	GCGAGCTCGACGAATACAC
VDrhlA3R	ACACCTGCACCGACACGTAG
VDrhlA1F	AGACCGTGCGCTACCTCGGCGAGCGGCTCAATTCCATCTGCTT
VDrhlA1R	GCTGCCGAGCCGGCGCACGTCGGCGGGGGTGGTGTATTCGTCGAGCTCC
promrhlAF	ACGTAGAATTCGGGAAAGCAGGCAGGGTAG
promrhlAR	ACGTAAAGCTTTTTCGATAGGCATGGCGTACTC

Transcriptional fusion strains of *B*. *glumae* were constructed by PCR amplification of a 923-bp fragment containing the promoter region of *rhlA* from BGR1 genome using primers promrhlAF and promrhlAR (Table 2). The PCR product was digested with *EcoR*I and *Hind*III and ligated within the corresponding sites in mini-CTX-*lux* [20]. The resulting construct was integrated into BGR1 and *tofI* ^*-*^ mutant chromosome at the *attB* site through conjugation. Successful chromosomal integration of construct in BGR1 and *tofI* ^*-*^ mutant was verified by PCR.

### LC/MS rhamnolipid analysis

The rhamnolipid concentration in the various bacterial cultures was determined by LC/MS [23]. During a period of six days, 400 μl culture samples were retrieved at regular time intervals and the OD_600_ was measured (Nanodrop ND-1000, Thermo Fisher Scientific). Then the samples were centrifuged at 16,000 × *g* for 10 min. to remove the bacteria. To 300 μl of supernatant, 300 μl acetonitrile containing 20 mg/L 5,6,7,8-tetradeutero-4-hydroxy-2-heptylquinoline (HHQ-d4) as the internal standard were added [12]. Samples were analyzed by high-performance liquid chromatography (HPLC; Waters 2795, Mississauga, ON, Canada) equipped with a C8 reverse-phase column (Kinetex, Phenomenex) using a water/acetonitrile gradient with a constant 2 mmol l^-1^ concentration of ammonium acetate [12]. The detector was a mass spectrometer (Quattro Premier XE, Waters). Analyses were carried out in the negative electrospray ionization (ESI-) mode.

### Measurement of *rhlA’-lux* expression

Expression from the *rhl* operon promoter in wild-type and QS-defective *tofI* ^*-*^ strains was quantified by measuring the luminescence of cultures of bacteria containing a transcriptional fusion of the *rhlA* promoter with the luciferase gene integrated in the genome. Cells from overnight cultures were washed twice in PBS and inoculated into 5 ml of liquid swarming medium at an initial OD_600_ = 0.05. The cultures were grown at 34°C with shaking (240 rpm) in a roller drum. At regular time intervals 200 μl culture samples were retrieved and the OD_600_ and luminescence (relative light units, RLU) were measured using a multimode plate reader (Cytation3, BioTek, Winooski, VT). The luminescence (*rhlA-lux* activity) corrected for culture density (RLU/OD_600_) was determined at the peak of *rhlA* expression after 10 h of incubation.

## Results

### Rhamnolipid production is necessary for *B*. *glumae* swarming motility

**Fig 1:**
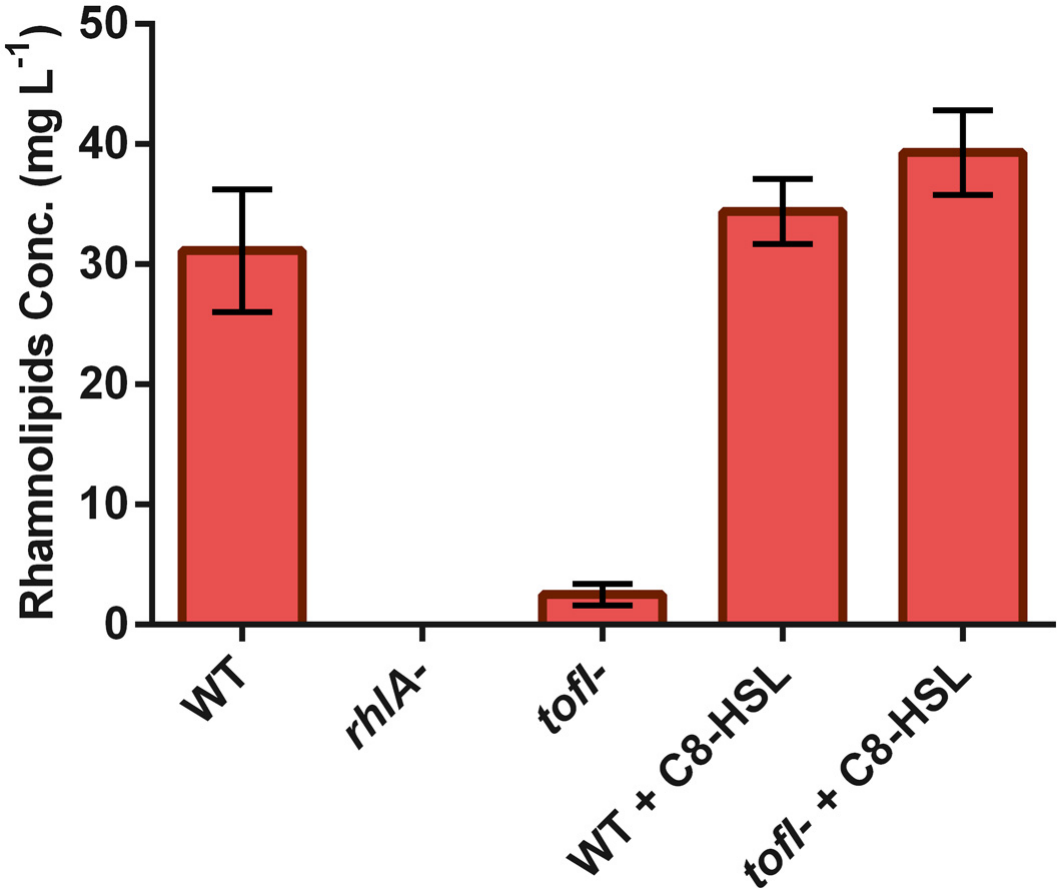
Rhamnolipids production of various *B*. *glumae* strains. Bacteria were grown in liquid swarming medium for 24 hours at 34°C. The mutant *rhlA* ^-^ is not capable of producing rhamnolipids. While the production of rhamnolipids by the *tofI* ^*-*^ mutant is almost abrogated, near wild-type production level are restored upon exogenous addition of 8 μM C_8_-HSL. The C_8_-HSL addition to wild-type does not significantly modify its rhamnolipid production. The error bars indicate the standard deviation of the mean for three independent cultures.

**Fig 2:**
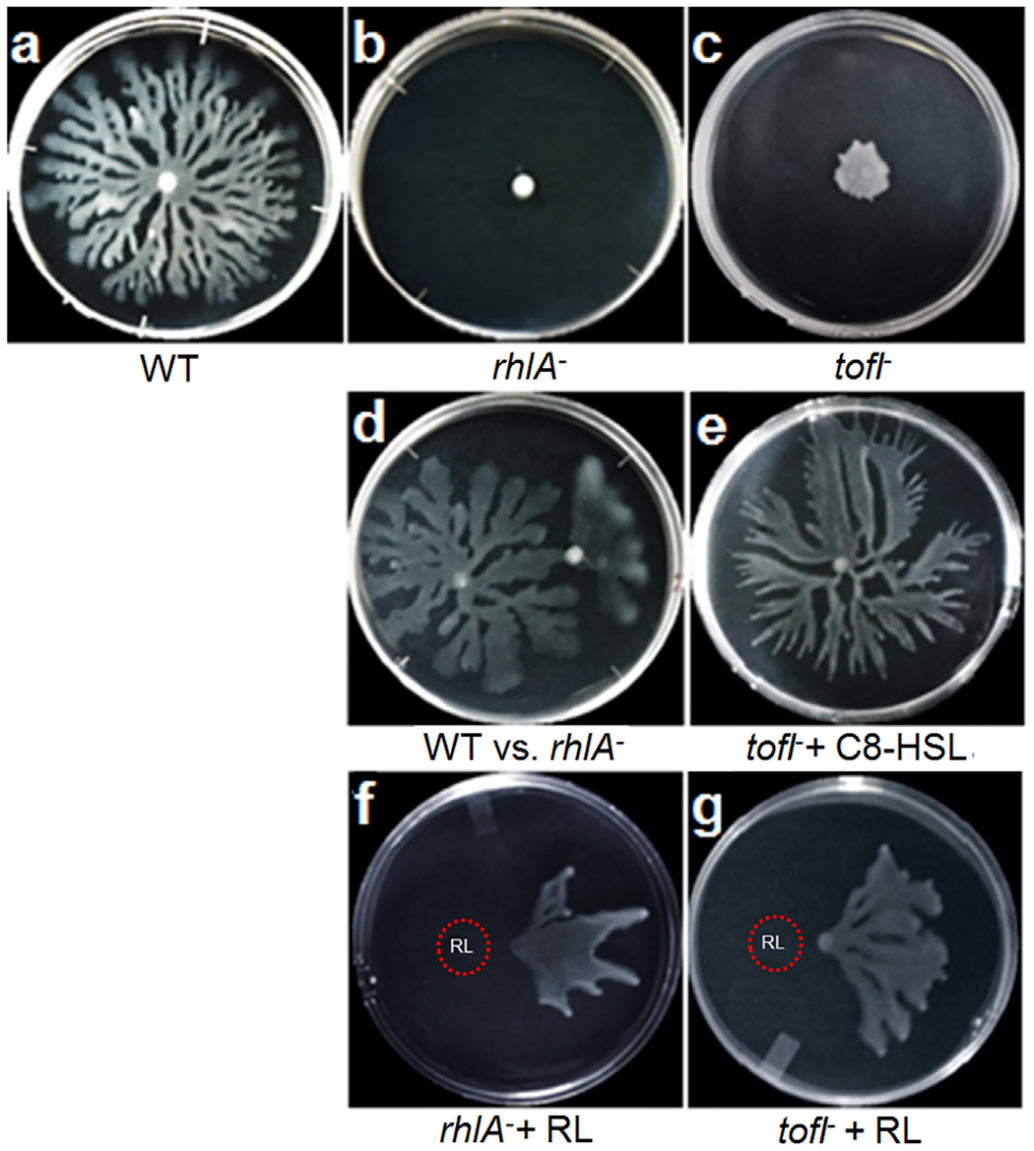
Swarming motilities of various *B*. *glumae* strains. Plates were incubated for 18 hours at 34°C. (a) Wild-type strain BGR1. (b) *rhlA* ^*-*^ mutant which is not capable to produce rhamnolipids completely loses its swarming phenotype. (c) Swarming of quorum sensing signal negative *tofI* ^*-*^ mutant is essentially abolished. (d) Wild-type strain (right) and *rhlA* ^-^ mutant (left) were placed on the same plate separated by a 2 cm distance between the inoculation points. The *rhlA* ^-^ mutant, which is incapable of swarming by itself, recovers its swarming phenotype by using the rhamnolipids diffusing from the wild-type strain colony. (e) Swarming motility of the *tofI* ^*-*^ mutant is recovered by exogenous addition of 8 μM C_8_-HSL to the swarming medium. (f) The *rhlA* ^-^ mutant recovers its swarming following exogenous supply of rhamnolipids, applied as a 5 μl drop of a 100 mg/ml rhamnolipids methanol solution on the surface of the swarming medium prior from inoculation at a 1 cm distance. (g) The addition of rhamnolipids to the swarming medium was performed like in (f), and similarly promotes the swarming of the *tofI* ^*-*^ mutant.

To verify whether rhamnolipids are involved in swarming motility of *B*. *glumae*, a *rhlA* ^-^ mutant was constructed in the wild-type strain BGR1 background and confirmed to have completely lost its ability to produce rhamnolipids (Fig 1). Investigating swarming motility showed that BGR1 exhibits a typical dendritic pattern formation, while the *rhlA* ^-^ mutant is unable to swarm (Fig 2a and 2b). We then examined whether the *rhlA* ^-^ mutant could recover its swarming phenotype by using the rhamnolipids produced by the wild-type strain. To do so, the *rhlA* ^-^ mutant and the wild-type strain were both inoculated on the same plate separated by distance of 2 cm between the inoculation points. The results show that the *rhlA* ^-^ mutant can exploit the rhamnolipids produced by the neighbouring wild-type colony and exhibit swarming (Fig 2d). Similarly, the *rhlA* ^-^ mutant could regain swarming motility when supplied with exogenous rhamnolipids: a drop of *B*. *glumae* rhamnolipids at 1 cm from the inoculation point of the *rhlA* ^-^ mutant was sufficient to induce swarming (Fig 2f), indicating that rhamnolipids are the only factor missing for swarming of that mutant, and likely the element provided by the WT colony in Fig 2d.

### Quorum sensing modulates swarming motility of *B*. *glumae* through regulation of rhamnolipid production

Earlier studies have demonstrated the lack of swarming motility in a QS signal-negative mutant (*tofI* ^-^) of strain BGR1 at both 28°C and 37°C [1]. While at 37°C the *tofI* ^-^ mutant is aflagellated, at 28°C the flagella are present and functional, leaving unexplained the non-swarming phenotype at 28°C. Since QS-controlled wetting agents are essential for swarming motility in other bacteria, we tested QS-dependent regulation of biosurfactant biosynthesis in *B*. *glumae* through examining the swarming motility of the wild-type strain BGR1 and *tofI* ^*-*^ mutant.

Swarming motility of the *tofI* ^*-*^ mutant was significantly reduced (Fig 2c) and the normal phenotype was restored by adding C_8_-HSL to the plates (Fig 2e). To investigate whether this impaired swarming motility is due to the lack of a surface wetting agent, we examined the production of rhamnolipids by the *tofI* ^*-*^ mutant grown in liquid culture, and measured a near-complete loss of production. Furthermore, exogenous addition of C_8_-HSL restored the production of rhamnolipids by the *tofI* ^*-*^ mutant to wild-type level (Fig 1).

**Fig 3:**
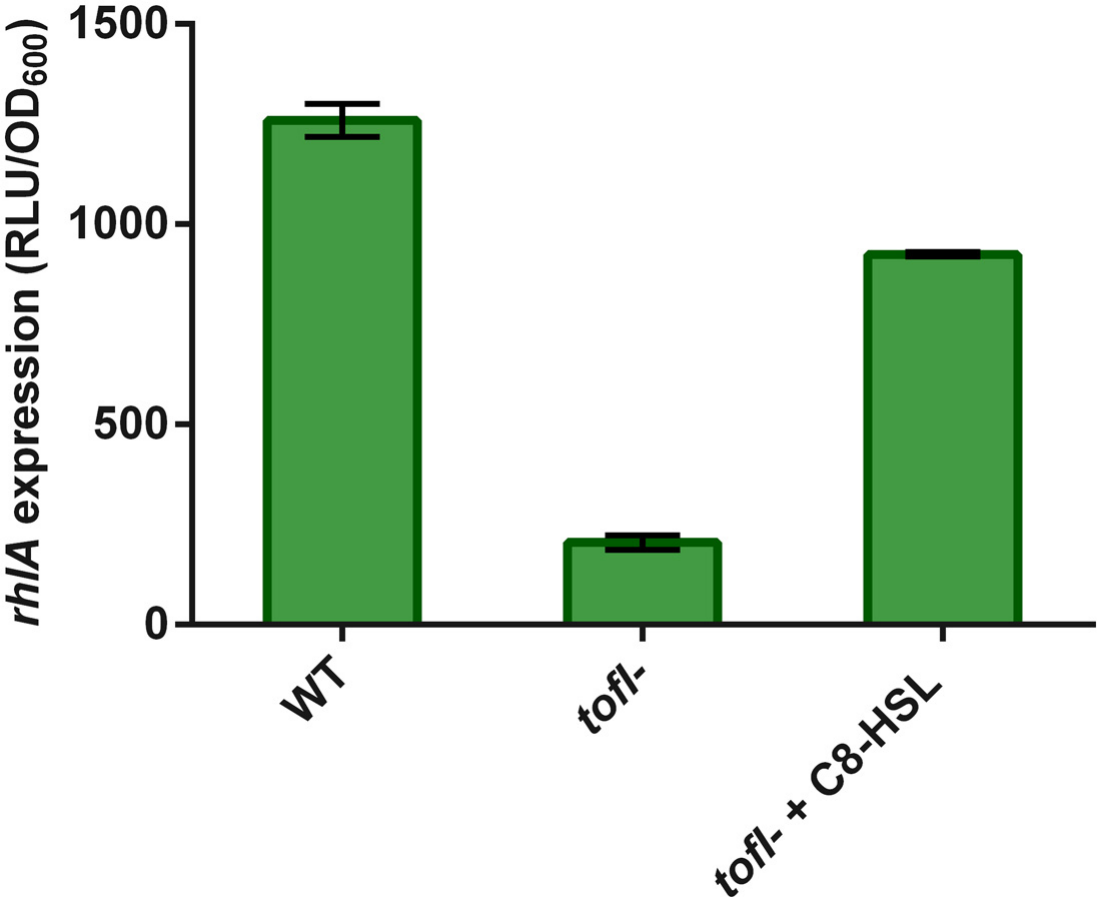
Expression from the *rhl* operon promoter in wild-type and *tofI* ^*-*^ strains carrying a chromosomal *rhlA’*-*lux* transcriptional reporter. Cultures were performed in liquid swarming medium cultures at 34°C for 10 h. The activity of *rhlA-lux* is significantly lower in the *tofI* ^*-*^ mutant than the wild-type strain and increases with the addition of 8 μM C_8_-HSL to the culture medium. The error bars indicate the standard deviation of the mean for three independent experiments.

Using a *rhlA*-*lux* reporter gene fusion, we found that transcription from the *rhl* operon promoter in the *tofI* ^*-*^ mutant was only 15% of wild-type levels, and was complemented by the exogenous addition of C_8_-HSL (Fig 3), demonstrating that QS positively regulates the expression of the *rhl* operon in *B*. *glumae*.

To verify whether the decreased swarming of the *tofI* ^*-*^ mutant is solely due to the decreased production of rhamnolipids, we tested the swarming of the *tofI* ^*-*^ with exogenous addition of rhamnolipids. Similar to the *rhlA-* mutant (Fig 2f), the *tofI* ^*-*^ mutant swarming defect was complemented by exogenous rhamnolipids (Fig 2g). Overall, these results demonstrate that rhamnolipids are positively regulated by QS in *B*. *glumae* and that QS directs swarming motility of *B*. *glumae* through rhamnolipids regulation.

**Fig 4:**
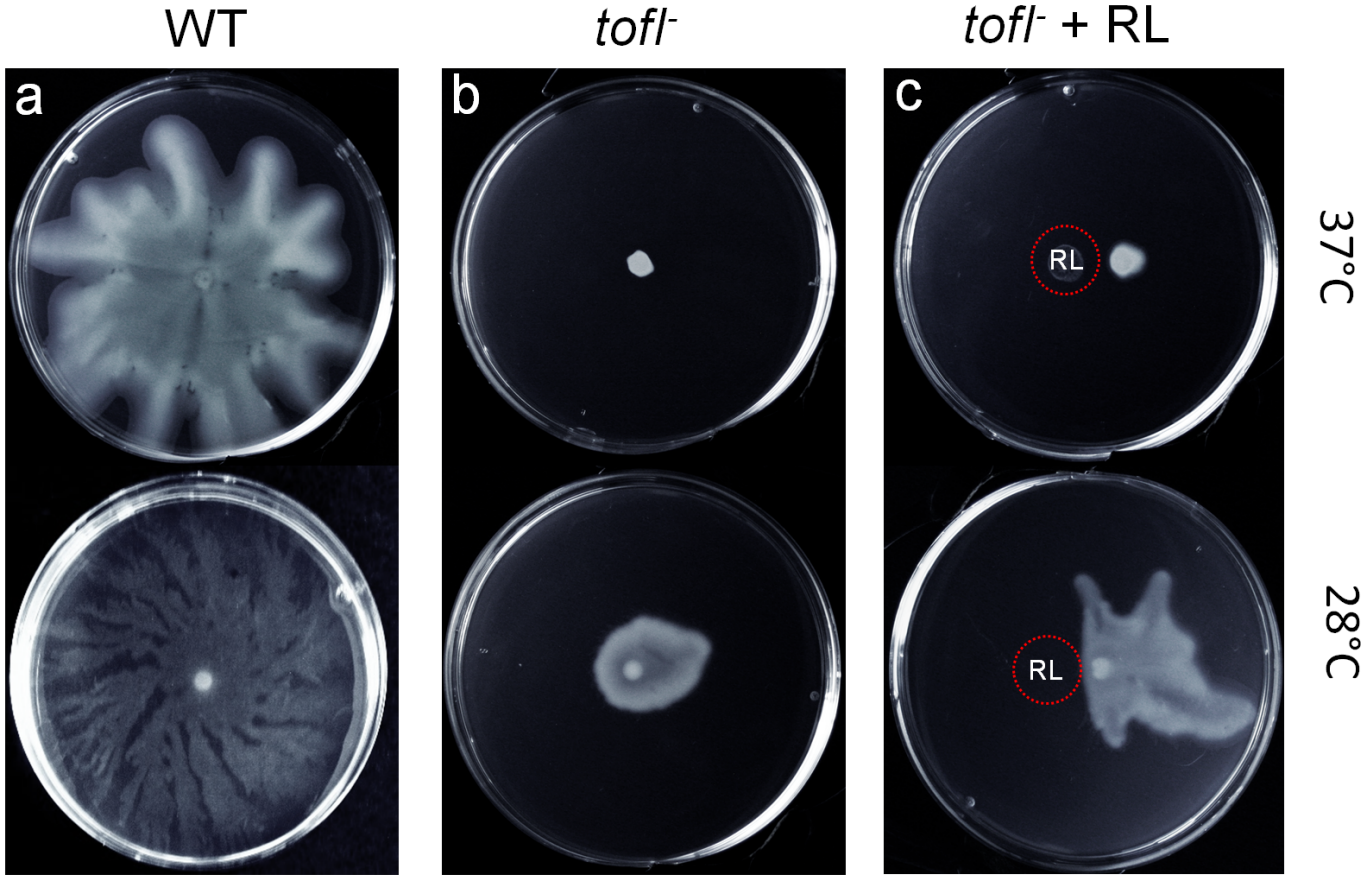
Swarming motility of *tofI* ^*-*^ mutant at 28°C and 37°C. (a) Wild-type strain BGR1 exhibits swarming at both temperatures, however it has larger/less defined tendrils than at 34°C. (b) No swarming of the *tofI* ^*-*^ mutant is observed at 37°C, and at 28°C it is nearly abolished, like at 34°C (Fig 2c). (c) While the *tofI* ^-^ mutant recovers its swarming upon exogenous addition of rhamnolipids at 28°C, the complementation is not achieved at 37°C.

**Fig 5:**
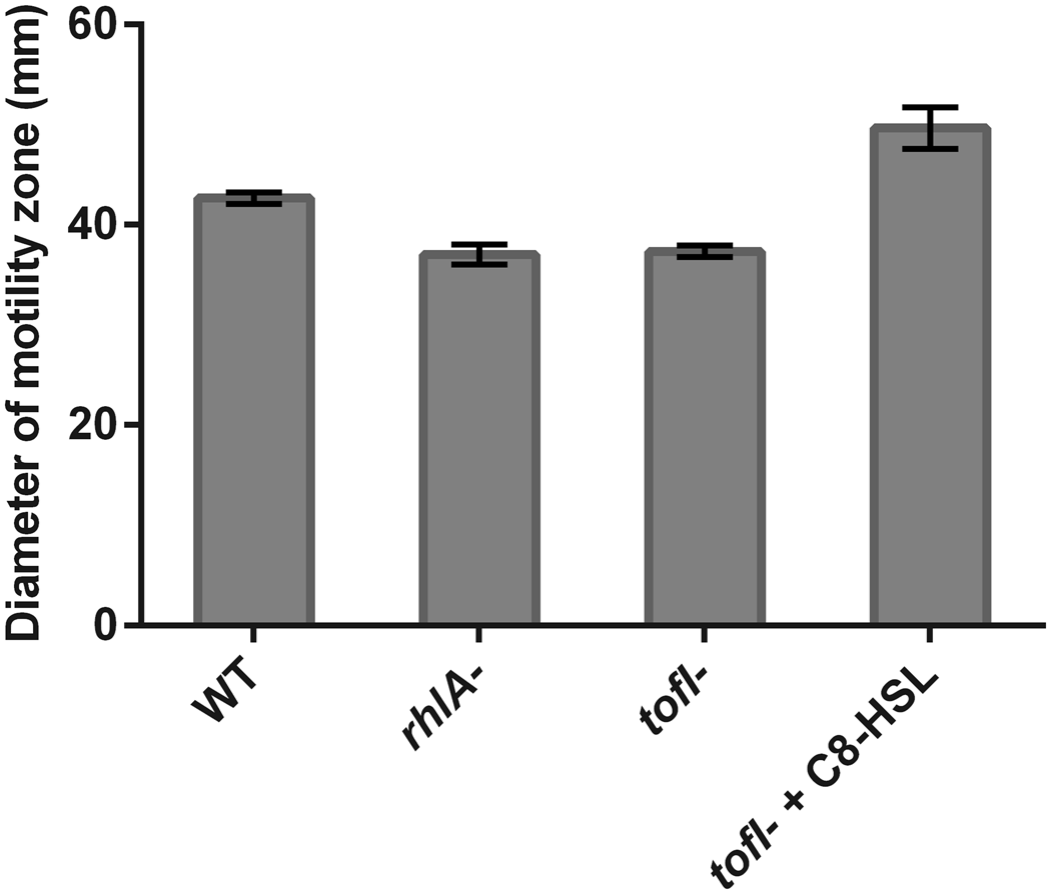
Swimming motility of *B*. *glumae* strains. Motility capacity was measured by diameter of motility zone on swimming agar plates at 34°C. The error bars indicate the standard deviation of the mean for three independent experiments.

As mentioned above, in *B*. *glumae* QS is required for the formation of flagella at 37°C, but not at 28°C [9]. Since we carried out our swarming assays at 34°C, the temperature found optimal for the development of well-defined *B*. *glumae* migrating tendrils, there was a possibility that lack of swarming of the *tofI* ^*-*^ mutant was due mainly to the absence of flagella, as 34°C is closer to 37°C than to 28°C. Thus, in order to ascertain that the abrogated swarming of the *tofI* ^*-*^ mutant (Fig 2c) is solely due to lack of rhamnolipids and not to defective flagella, we also compared the swarming of *tofI* ^*-*^ mutant at 28°C and 37°C. In accordance with results from Kim *et al*. (2007), the *tofI* ^*-*^ mutant was unable to swarm at 37°C, and at 28°C its swarming was essentially the same than at 34°C (Fig 4a and 4b). Accordingly, the *tofI* ^*-*^ mutant swarming defect at 28°C was restored by exogenous rhamnolipids but not at 37°C (Fig 4C). Furthermore, we examined flagella-driven swimming motility of the *tofI* ^*-*^ and *rhlA* ^*-*^mutants at 34°C. In line with the data shown in Fig 2g indicating that impaired swarming of the *tofI* ^*-*^ mutant is merely due to the lack of rhamnolipid biosynthesis, swimming motility in the *tofI* ^*-*^ mutant was similar to that of the wild-type strain (Fig 5).

## Discussion

Similar to *B*. *pseudomallei*, *B*. *thailandensis* and *P*. *aeruginosa*, *B*. *glumae* also produces extracellular surface-active rhamnolipids. Over the last two decades, the regulation of rhamnolipids *via* QS and their role in swarming motility and as virulence factors have been extensively characterized in *P*. *aeruginosa* [11,24,25]. However, these biosurfactants have been much less investigated in *Burkholderia* species. Previously, we have reported a role for rhamnolipids in the swarming motility of *B*. *thailandensis* [12]. However, no study had yet addressed the function and regulation of rhamnolipids in the phytopathogen *B*. *glumae*.

We have confirmed that rhamnolipids are necessary for swarming of *B*. *glumae* by generating a rhamnolipid-negative *rhlA* ^*-*^ mutant in the wild-type BGR1 model strain background and by complementing with rhamnolipids provided either by the wild-type strain or added exogenously to the medium.

Since in *P*. *aeruginosa* QS controls the expression of the *rhlAB* operon, responsible for rhamnolipid biosynthesis, we hypothesized that a similar regulatory system directs rhamnolipid biosynthesis in *B*. *glumae*. Indeed, we found that rhamnolipids are positively regulated by QS in this bacterium, adding this biosurfactant to the list of QS-dependent factors in *B*. *glumae*. Considering the dual regulation of polar flagellum genes and flagellar morphogenesis by QS and temperature in *B*. *glumae* [1,9], our finding is important to understand the mechanistic role of QS in regulation of factors contributing to swarming motility.

Previous studies have demonstrated that, while at 37°C the wild-type strain is capable of swimming and swarming motility, both of these motility phenotypes are strongly reduced in the *tofI* ^*-*^ mutant [1]. On the other hand, a QS-deficient mutant of strain BGR1 grown at 28°C retains its flagella and regains swimming, but is still incapable of swarming [1]. We thus reasoned that a swarming motility defect at lower temperatures could be due to lack of wetting agent production. A similar phenomenon was observed for *Burkholderia cepacia* and evidence was provided that its swarming motility might be controlled through QS-dependent regulation of biosurfactant biosynthesis [26]. Our results of normal swimming and defective swarming of the *tofI* ^*-*^ mutant at 34°C that can be complemented with rhamnolipids demonstrate that at a temperature where QS does not regulate flagellar morphogenesis, it controls swarming motility solely through rhamnolipid production. However, at higher temperatures such as 37°C, where flagellar biosynthesis is under control of QS, the *tofI* ^*-*^ mutant is aflagellated and hence exhibits no swarming even if the rhamnolipids are being provided exogenously. Furthermore, with regards to the swarming of the *tofI* ^*-*^ mutant at 28°C and 37°C, we can conclude that at 34°C the flagellar morphogenesis and swarming phenotype are similar to those at 28°C.

Many factors regulated by QS in *B*. *glumae* are virulence determinants (e.g. toxoflavin, flagella). Motility is important for virulence and there are many reports that non-motile strains of phytopathogenic bacteria are avirulent [1,27]; furthermore rhamnolipids contribute to the virulence of *P*. *aeruginosa* and *B*. *pseudomallei* [28,29]. Taken together, rhamnolipids might well contribute to the virulence of *B*. *glumae* through promotion of swarming motility.

It has been proposed that when environmental conditions (e.g. nutrient availability) are becoming less suitable for the bacteria, swarming motility promotes their dispersion towards finding new niches [30]. Since rhamnolipids are costly metabolites to produce, we can envisage that in a phytopathogenic bacterium such as *B*. *glumae* the regulation of rhamnolipids by QS enhances the efficacy of rhamnolipids by balancing their benefits to their cost of production in situations where they contribute the most to the survival and fitness [31].

Overall, our results demonstrate that rhamnolipids production is required for swarming motility of *B*. *glumae*, which could reflect a possible role in pathogenicity and survival in the host tissue. Therefore, elucidating the exact function of rhamnolipids in swarming motility and bacterial dispersal, combined with pathogenicity tests with the *rhlA* ^*-*^ mutant may provide insight into future strategies for controlling *B*. *glumae* virulence.
